# (*Z*)-2-(4-Nitro­benzyl­idene)-1-benzofuran-3(2*H*)-one

**DOI:** 10.1107/S1600536811041869

**Published:** 2011-10-12

**Authors:** J. Satyanarayana Reddy, N. Ravikumar, J. Venkata Prasad, G. Gopi Krishna, K. Anand Solomon

**Affiliations:** a Sankar Foundation Research Institute, Naiduthota, Vepagunta, Visakhapatnam, Andhra pradesh 530 047, India

## Abstract

In the crystal structure of the title compound, C_15_H_9_NO_4_, weak C—H⋯O inter­actions generate rings with *R* ^2^_2_(8) motifs. The supra­molecular aggregation is completed by the presence of C—H⋯O and van der Waals inter­actions.

## Related literature

For the synthesis and biological activity of substituted aurones, see: Varma & Varma (1992 ▶); Beney *et al.* (2001 ▶); Sim *et al.* (2008 ▶). For the assignment of conformations and the orientation of the substituents, see: Nardelli (1983 ▶, 1995 ▶); Klyne & Prelog (1960 ▶). For hydrogen bonds, see: Desiraju & Steiner (1999 ▶). For graph-set analysis of hydrogen bonds, see: Etter *et al.* (1990 ▶); Bernstein *et al.* (1995 ▶). For the diverse theraputic properties of aurones, see: Villemin *et al.* (1998 ▶). Several multifunctionalized aurones have been reported to exhibit anti-malarial (Souard *et al.* 2010 ▶) and anti-histamine (Wang *et al.* 2007 ▶) properties. 
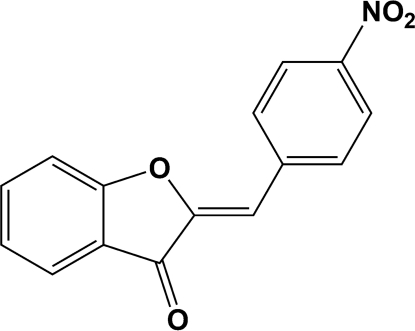


## Experimental

### 

#### Crystal data

C_15_H_9_NO_4_
*M* *_r_* = 267.23
Triclinic, 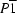

*a* = 6.6916 (2) Å
*b* = 7.4708 (2) Å
*c* = 12.6414 (3) Å
α = 100.459 (1)°
β = 93.019 (2)°
γ = 102.043 (1)°
*V* = 605.09 (3) Å^3^
*Z* = 2
Mo *K*α radiation
μ = 0.11 mm^−1^
*T* = 303 K
0.30 × 0.20 × 0.20 mm

#### Data collection

Bruker Kappa APEXII CCD diffractometer
Absorption correction: multi-scan (*SADABS*; Bruker, 2004) ▶ *T*_min_ = 0.932, *T*_max_ = 0.955
12519 measured reflections
2116 independent reflections
1869 reflections with *I* > 2σ(*I*)
*R*_int_ = 0.020

#### Refinement

*R*[*F* ^2^ > 2σ(*F* ^2^)] = 0.037
*wR*(*F* ^2^) = 0.105
*S* = 1.03
2116 reflections
181 parameters
H-atom parameters constrained
Δρ_max_ = 0.20 e Å^−3^
Δρ_min_ = −0.25 e Å^−3^

### 

Data collection: *APEX2* (Bruker, 2004 ▶); cell refinement: *APEX2* and *SAINT* (Bruker, 2004 ▶); data reduction: *SAINT* and *XPREP* (Bruker, 2004 ▶); program(s) used to solve structure: *SIR92* (Altomare *et al.*, 1993 ▶); program(s) used to refine structure: *SHELXL97* (Sheldrick, 2008 ▶); molecular graphics: *ORTEP-3* (Farrugia, 1997 ▶) and *Mercury* (Macrae *et al.*, 2006 ▶); software used to prepare material for publication: *SHELXL97*.

## Supplementary Material

Crystal structure: contains datablock(s) I, global. DOI: 10.1107/S1600536811041869/zj2025sup1.cif

Structure factors: contains datablock(s) I. DOI: 10.1107/S1600536811041869/zj2025Isup2.hkl

Supplementary material file. DOI: 10.1107/S1600536811041869/zj2025Isup3.cml

Additional supplementary materials:  crystallographic information; 3D view; checkCIF report

## Figures and Tables

**Table 1:** Hydrogen-bond geometry (Å, °)

*D*—H⋯*A*	*D*—H	H⋯*A*	*D*⋯*A*	*D*—H⋯*A*
C15—H15⋯O1	0.93	2.32	2.9547 (16)	125
C9—H9⋯O2^i^	0.93	2.50	3.2951 (14)	143

Symmetry code: (i) 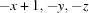.

**Fig. 1.**
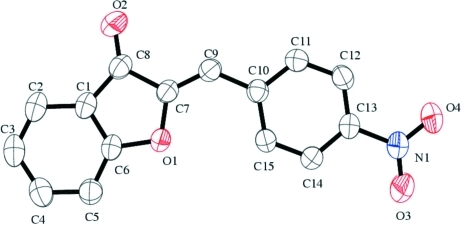
ORTEP diagram of (Z)-2-(4-nitrobenzylidene)benzofuran-3(2H)-one. (Thermal ellipsoids are at 50% probability level).

**Fig. 2.**
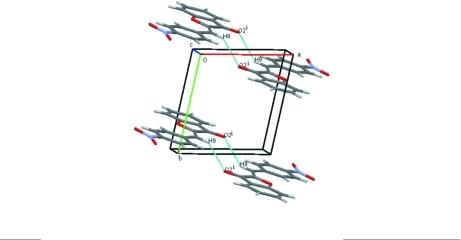
Crystal packing diagram of the title compound.

**Fig. 3.**
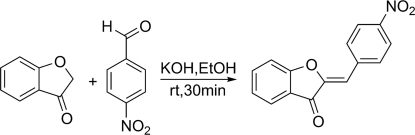
The synthetic scheme of the title compound.

## supplementary crystallographic information

### Comment

Aurones belong flavonoids family, which are structurally isomers of flavones. They form essential structural scaffolds in many natural and synthetic molecules possessing diverse therapeutic properties (Villemin *et al.* 1998) Several multifunctionalized aurones were reported to exhibit anti-malarial (Souard *et al.* 2010) and anti-histamine (Wang *et al.* 2007) properties.

The title compound (Fig.1),*C*_15_H_9_NO_4_,crystallized in triclinic space group P-1 with two molecules in the assymetric unit (Fig.2).The crystal structure of (I) is stabilized by C—H···O interactions.The range of H···O distances (Table 1) found in (I) agrees with those found for C—H···O hydrogen bonds (Desiraju & Steiner,1999). The coumaranone moiety at C10 is in co-planar conformation [C7—C8—C9—C10=179.55 (13)°].The translational related molecules interact with each other *via* weak C—H···O [C9—H9···O2: H9···O2 = 2.50 Å, θ = 143°] hydrogen bonds along the *c* axis, and form a one dimensional chain (Fig. 3).

### Experimental

3-coumaranone was allowed to react with 4-nitrobenzaldehyde in ethanolic solution of potassium hydroxide for 30 minutes to yield the title compound (Fig.4). The pure product was obtained by recrystallization in methanol.

### Refinement

Refinement of F^2^ against ALL reflections. The weighted *R*-factor *wR* and goodness of fit S are based on F^2^, conventional *R*-factors *R* are based on F, with F set to zero for negative F^2^. The threshold expression of F^2^ > 2sigma(F^2^) is used only for calculating *R*-factors(gt) *etc*. and is not relevant to the choice of reflections for refinement. *R*-factors based on F^2^ are statistically about twice as large as those based on F, and R– factors based on ALL data will be even larger.

### Crystal data

**Table:**

C_15_H_9_NO_4_	*Z* = 2
*M**_r_* = 267.23	*F*(000) = 276
Triclinic, *P*1	*D*_x_ = 1.467 Mg m^−^^3^
Hall symbol: -P 1	Melting point: 460 K
*a* = 6.6916 (2) Å	Mo *K*α radiation, λ = 0.71073 Å
*b* = 7.4708 (2) Å	Cell parameters from 7266 reflections
*c* = 12.6414 (3) Å	θ = 2.8–30.5°
α = 100.459 (1)°	µ = 0.11 mm^−^^1^
β = 93.019 (2)°	*T* = 303 K
γ = 102.043 (1)°	Block, yellow
*V* = 605.09 (3) Å^3^	0.30 × 0.20 × 0.20 mm

### Data collection

**Table:**

Bruker Kappa APEXII CCD diffractometer	2116 independent reflections
Radiation source: fine-focus sealed tube	1869 reflections with *I* > 2σ(*I*)
graphite	*R*_int_ = 0.020
ω and φ scans	θ_max_ = 25.0°, θ_min_ = 2.8°
Absorption correction: multi-scan (*SADABS*; Bruker, 2004)	*h* = −7→7
*T*_min_ = 0.932, *T*_max_ = 0.955	*k* = −8→8
12519 measured reflections	*l* = −15→15

### Refinement

**Table:**

Refinement on *F*^2^	Primary atom site location: structure-invariant direct methods
Least-squares matrix: full	Secondary atom site location: difference Fourier map
*R*[*F*^2^ > 2σ(*F*^2^)] = 0.037	Hydrogen site location: inferred from neighbouring sites
*wR*(*F*^2^) = 0.105	H-atom parameters constrained
*S* = 1.03	*w* = 1/[σ^2^(*F*_o_^2^) + (0.0599*P*)^2^ + 0.1178*P*] where *P* = (*F*_o_^2^ + 2*F*_c_^2^)/3
2116 reflections	(Δ/σ)_max_ < 0.001
181 parameters	Δρ_max_ = 0.20 e Å^−^^3^
0 restraints	Δρ_min_ = −0.25 e Å^−^^3^

### Special details

**Table:**

Geometry. All e.s.d.'s (except the e.s.d. in the dihedral angle between two l.s. planes) are estimated using the full covariance matrix. The cell e.s.d.'s are taken into account individually in the estimation of e.s.d.'s in distances, angles and torsion angles; correlations between e.s.d.'s in cell parameters are only used when they are defined by crystal symmetry. An approximate (isotropic) treatment of cell e.s.d.'s is used for estimating e.s.d.'s involving l.s. planes.
Refinement. Refinement of *F*^2^ against ALL reflections. The weighted *R*-factor *wR* and goodness of fit *S* are based on *F*^2^, conventional *R*-factors *R* are based on *F*, with *F* set to zero for negative *F*^2^. The threshold expression of *F*^2^ > σ(*F*^2^) is used only for calculating *R*-factors(gt) *etc*. and is not relevant to the choice of reflections for refinement. *R*-factors based on *F*^2^ are statistically about twice as large as those based on *F*, and *R*- factors based on ALL data will be even larger.

### Fractional atomic coordinates and isotropic or equivalent isotropic displacement parameters (Å^2^)

**Table:**

	*x*	*y*	*z*	*U*_iso_*/*U*_eq_	
C1	0.8384 (2)	0.28398 (18)	−0.19093 (11)	0.0456 (3)	
C2	0.7837 (2)	0.3025 (2)	−0.29555 (11)	0.0548 (4)	
H2	0.6469	0.2729	−0.3234	0.066*	
C3	0.9366 (3)	0.3652 (2)	−0.35600 (12)	0.0595 (4)	
H3	0.9037	0.3790	−0.4260	0.071*	
C4	1.1407 (3)	0.4087 (2)	−0.31413 (12)	0.0586 (4)	
H4	1.2417	0.4519	−0.3569	0.070*	
C5	1.1987 (2)	0.3898 (2)	−0.21071 (12)	0.0526 (4)	
H5	1.3355	0.4179	−0.1830	0.063*	
C6	1.0423 (2)	0.32720 (17)	−0.15171 (10)	0.0431 (3)	
C7	0.88073 (19)	0.22680 (18)	−0.01762 (11)	0.0429 (3)	
C8	0.7214 (2)	0.21929 (19)	−0.10621 (12)	0.0485 (3)	
C9	0.8470 (2)	0.17046 (18)	0.07516 (11)	0.0452 (3)	
H9	0.7102	0.1217	0.0832	0.054*	
C10	0.9913 (2)	0.17343 (17)	0.16591 (10)	0.0411 (3)	
C11	0.9136 (2)	0.11596 (19)	0.25736 (11)	0.0470 (3)	
H11	0.7727	0.0733	0.2574	0.056*	
C12	1.0401 (2)	0.12085 (19)	0.34749 (11)	0.0477 (3)	
H12	0.9866	0.0829	0.4083	0.057*	
C13	1.2473 (2)	0.18320 (18)	0.34544 (10)	0.0444 (3)	
C14	1.3322 (2)	0.23746 (19)	0.25581 (11)	0.0475 (3)	
H14	1.4736	0.2769	0.2562	0.057*	
C15	1.2041 (2)	0.23210 (18)	0.16603 (11)	0.0452 (3)	
H15	1.2592	0.2677	0.1050	0.054*	
N1	1.3855 (2)	0.19263 (19)	0.44088 (10)	0.0575 (3)	
O1	1.07162 (13)	0.29789 (13)	−0.04757 (7)	0.0459 (3)	
O2	0.53638 (16)	0.16865 (18)	−0.10414 (10)	0.0732 (4)	
O3	1.5647 (2)	0.2687 (3)	0.44252 (12)	0.1051 (6)	
O4	1.31586 (19)	0.12304 (19)	0.51458 (9)	0.0760 (4)	

### Atomic displacement parameters (Å^2^)

**Table:**

	*U*^11^	*U*^22^	*U*^33^	*U*^12^	*U*^13^	*U*^23^
C1	0.0521 (8)	0.0404 (7)	0.0431 (7)	0.0118 (6)	−0.0073 (6)	0.0070 (5)
C2	0.0648 (9)	0.0512 (8)	0.0466 (8)	0.0140 (7)	−0.0133 (7)	0.0092 (6)
C3	0.0845 (11)	0.0537 (8)	0.0392 (7)	0.0150 (8)	−0.0068 (7)	0.0108 (6)
C4	0.0747 (10)	0.0542 (8)	0.0465 (8)	0.0093 (7)	0.0062 (7)	0.0142 (6)
C5	0.0543 (8)	0.0539 (8)	0.0483 (8)	0.0072 (6)	0.0001 (6)	0.0135 (6)
C6	0.0513 (8)	0.0395 (7)	0.0382 (7)	0.0108 (5)	−0.0043 (5)	0.0084 (5)
C7	0.0404 (7)	0.0435 (7)	0.0439 (7)	0.0085 (5)	−0.0025 (5)	0.0090 (5)
C8	0.0443 (8)	0.0499 (8)	0.0513 (8)	0.0110 (6)	−0.0058 (6)	0.0121 (6)
C9	0.0406 (7)	0.0476 (7)	0.0468 (8)	0.0085 (5)	0.0004 (6)	0.0102 (6)
C10	0.0443 (7)	0.0385 (6)	0.0405 (7)	0.0092 (5)	0.0016 (5)	0.0084 (5)
C11	0.0424 (7)	0.0525 (8)	0.0481 (8)	0.0108 (6)	0.0064 (6)	0.0143 (6)
C12	0.0537 (8)	0.0522 (8)	0.0414 (7)	0.0143 (6)	0.0092 (6)	0.0159 (6)
C13	0.0511 (8)	0.0451 (7)	0.0378 (7)	0.0124 (6)	−0.0011 (6)	0.0098 (5)
C14	0.0418 (7)	0.0547 (8)	0.0450 (8)	0.0055 (6)	−0.0003 (6)	0.0145 (6)
C15	0.0463 (7)	0.0510 (7)	0.0385 (7)	0.0067 (6)	0.0023 (5)	0.0145 (6)
N1	0.0585 (8)	0.0713 (8)	0.0436 (7)	0.0123 (6)	−0.0027 (6)	0.0187 (6)
O1	0.0426 (5)	0.0546 (5)	0.0400 (5)	0.0067 (4)	−0.0041 (4)	0.0154 (4)
O2	0.0437 (6)	0.1012 (9)	0.0775 (8)	0.0093 (6)	−0.0083 (5)	0.0363 (7)
O3	0.0651 (8)	0.1611 (15)	0.0821 (9)	−0.0170 (9)	−0.0255 (7)	0.0637 (10)
O4	0.0768 (8)	0.1133 (10)	0.0450 (6)	0.0215 (7)	0.0047 (5)	0.0342 (6)

### Geometric parameters (Å, °)

**Table:**

C1—C6	1.3780 (19)	C9—C10	1.4541 (18)
C1—C2	1.3922 (19)	C9—H9	0.9300
C1—C8	1.453 (2)	C10—C11	1.3935 (19)
C2—C3	1.365 (2)	C10—C15	1.3993 (19)
C2—H2	0.9300	C11—C12	1.3736 (19)
C3—C4	1.388 (2)	C11—H11	0.9300
C3—H3	0.9300	C12—C13	1.371 (2)
C4—C5	1.384 (2)	C12—H12	0.9300
C4—H4	0.9300	C13—C14	1.3816 (19)
C5—C6	1.369 (2)	C13—N1	1.4628 (18)
C5—H5	0.9300	C14—C15	1.3752 (19)
C6—O1	1.3837 (16)	C14—H14	0.9300
C7—C9	1.331 (2)	C15—H15	0.9300
C7—O1	1.3777 (16)	N1—O3	1.2127 (17)
C7—C8	1.4900 (18)	N1—O4	1.2150 (17)
C8—O2	1.2200 (17)		
			
C6—C1—C2	119.87 (14)	C7—C9—H9	115.1
C6—C1—C8	106.79 (12)	C10—C9—H9	115.1
C2—C1—C8	133.31 (13)	C11—C10—C15	118.52 (12)
C3—C2—C1	118.13 (14)	C11—C10—C9	118.28 (12)
C3—C2—H2	120.9	C15—C10—C9	123.20 (12)
C1—C2—H2	120.9	C12—C11—C10	121.54 (13)
C2—C3—C4	120.73 (14)	C12—C11—H11	119.2
C2—C3—H3	119.6	C10—C11—H11	119.2
C4—C3—H3	119.6	C13—C12—C11	118.23 (13)
C5—C4—C3	122.12 (15)	C13—C12—H12	120.9
C5—C4—H4	118.9	C11—C12—H12	120.9
C3—C4—H4	118.9	C12—C13—C14	122.37 (13)
C6—C5—C4	115.94 (14)	C12—C13—N1	119.42 (12)
C6—C5—H5	122.0	C14—C13—N1	118.21 (13)
C4—C5—H5	122.0	C15—C14—C13	118.91 (13)
C5—C6—C1	123.20 (13)	C15—C14—H14	120.5
C5—C6—O1	123.91 (12)	C13—C14—H14	120.5
C1—C6—O1	112.89 (12)	C14—C15—C10	120.40 (13)
C9—C7—O1	124.72 (12)	C14—C15—H15	119.8
C9—C7—C8	126.03 (13)	C10—C15—H15	119.8
O1—C7—C8	109.24 (11)	O3—N1—O4	122.99 (13)
O2—C8—C1	130.02 (13)	O3—N1—C13	118.44 (13)
O2—C8—C7	125.87 (14)	O4—N1—C13	118.57 (13)
C1—C8—C7	104.10 (11)	C7—O1—C6	106.90 (10)
C7—C9—C10	129.88 (13)		
			
C6—C1—C2—C3	−0.5 (2)	C7—C9—C10—C11	176.04 (13)
C8—C1—C2—C3	−178.41 (14)	C7—C9—C10—C15	−3.6 (2)
C1—C2—C3—C4	0.2 (2)	C15—C10—C11—C12	1.8 (2)
C2—C3—C4—C5	0.4 (2)	C9—C10—C11—C12	−177.82 (12)
C3—C4—C5—C6	−0.6 (2)	C10—C11—C12—C13	−0.4 (2)
C4—C5—C6—C1	0.2 (2)	C11—C12—C13—C14	−1.2 (2)
C4—C5—C6—O1	179.20 (12)	C11—C12—C13—N1	179.06 (12)
C2—C1—C6—C5	0.3 (2)	C12—C13—C14—C15	1.2 (2)
C8—C1—C6—C5	178.73 (12)	N1—C13—C14—C15	−179.02 (12)
C2—C1—C6—O1	−178.73 (11)	C13—C14—C15—C10	0.3 (2)
C8—C1—C6—O1	−0.35 (15)	C11—C10—C15—C14	−1.8 (2)
C6—C1—C8—O2	178.90 (15)	C9—C10—C15—C14	177.86 (12)
C2—C1—C8—O2	−3.0 (3)	C12—C13—N1—O3	−171.28 (15)
C6—C1—C8—C7	−1.28 (14)	C14—C13—N1—O3	9.0 (2)
C2—C1—C8—C7	176.79 (14)	C12—C13—N1—O4	9.1 (2)
C9—C7—C8—O2	3.7 (2)	C14—C13—N1—O4	−170.61 (13)
O1—C7—C8—O2	−177.66 (13)	C9—C7—O1—C6	175.88 (12)
C9—C7—C8—C1	−176.09 (13)	C8—C7—O1—C6	−2.75 (13)
O1—C7—C8—C1	2.52 (14)	C5—C6—O1—C7	−177.09 (12)
O1—C7—C9—C10	2.1 (2)	C1—C6—O1—C7	1.98 (14)
C8—C7—C9—C10	−179.55 (13)		

### Hydrogen-bond geometry (Å, °)

**Table:**

*D*—H···*A*	*D*—H	H···*A*	*D*···*A*	*D*—H···*A*
C15—H15···O1	0.93	2.32	2.9547 (16)	125
C9—H9···O2^i^	0.93	2.50	3.2951 (14)	143

Symmetry codes: (i) −*x*+1, −*y*, −*z*.

## References

[bb1] Altomare, A., Cascarano, G., Giacovazzo, C. & Guagliardi, A. (1993). *J. Appl. Cryst.* **26**, 343–350.

[bb2] Beney, C., Mariotte, A. M. & Boumendjel, A. (2001). *Heterocycles*, **55**, 967–972.

[bb3] Bernstein, J., Davis, R. E., Shimoni, L. & Chang, N.-L. (1995). *Angew. Chem. Int. Ed. Engl.* **34**, 1555–1573.

[bb4] Bruker (2004). *APEX2*, *SAINT*, *XPREP* and *SADABS* Bruker AXS Inc., Madison, Wisconsin, USA.

[bb5] Desiraju, G. A. & Steiner, T. (1999). *The Weak Hydrogen Bond in Structural Chemistry and Biology* New York: Oxford University Press Inc.

[bb6] Etter, M. C., MacDonald, J. C. & Bernstein, J. (1990). *Acta Cryst.* B**46**, 256–262. 10.1107/s0108768189012929 2344397

[bb7] Farrugia, L. J. (1997). *J. Appl. Cryst.* **30**, 565.

[bb8] Klyne, W. & Prelog, V. (1960). *Experientia*, **16**, 521–568.

[bb9] Macrae, C. F., Edgington, P. R., McCabe, P., Pidcock, E., Shields, G. P., Taylor, R., Towler, M. & van de Streek, J. (2006). *J. Appl. Cryst.* **39**, 453–457.

[bb10] Nardelli, M. (1983). *Acta Cryst.* C**39**, 1141–1142.

[bb11] Nardelli, M. (1995). *J. Appl. Cryst.* **28**, 659.

[bb12] Sheldrick, G. M. (2008). *Acta Cryst.* A**64**, 112–122. 10.1107/S0108767307043930 18156677

[bb13] Sim, H. M., Lee, C. Y., Ee, P. L. & Go, M. L. (2008). *Eur. J. Pharm. Sci.* **35**, 293–306. 10.1016/j.ejps.2008.07.008 18725288

[bb14] Souard, F., Okombi, S., Beney, C., Chevalley, S., Valentin, A. & Boumendjel, A. (2010). *Bioorg. Med. Chem.* **1**, 5724–5731. 10.1016/j.bmc.2010.06.008 20630767

[bb15] Varma, R. S. & Varma, M. (1992). *Tetrahedron Lett.* **33**, 5937–5940.

[bb16] Villemin, D., Martin, B. & Bar, N. (1998). *Molecules*, **3**, 88–93.

[bb17] Wang, J., Wang, N., Yao, X. & Kitanaka, S. (2007). *J. Trad. Med*, **2**, 23–29.

